# Complete genome sequences of metal-oxidizing bacteria *Leptothrix mechoopdaensis* BB-3 and BB-4

**DOI:** 10.1128/mra.01458-25

**Published:** 2026-05-28

**Authors:** Emily J. Fleming, Abigail McIntyre, Gracee K. Tothero, Joyce A. Zimmer, Jessica L. Keffer, Lydia N. Pierce, Kathleen R. Beilsmith, Rosemarie Wilton, Clara S. Chan

**Affiliations:** 1 Department of Biological Sciences, California State University 14663, Chico, California, USA; 2 Microbiology Graduate Program, University of Delaware 5972, Newark, Delaware, USA; 3 Department of Earth Sciences, University of Delaware 5972, Newark, Delaware, USA; 4 Argonne National Laboratory 1291, Lemont, Illinois, USA; 5 University of Tennessee 4292, Knoxville, Tennessee, USA; 6 University of Chicago 2462, Chicago, Illinois, USA; University of Wisconsin-Madison, Madison, Wisconsin, USA

**Keywords:** *Leptothrix*, iron oxidizers

## Abstract

*Leptothrix mechoopdaensis* BB-3 and BB-4 are metal-oxidizing, sheathed, filamentous bacteria that were isolated from a woodland iron seep near Centerville, Chico, CA, USA. Here, we report a closed circular genome sequence for each strain, assembled from reads sequenced on an Oxford Nanopore FLO-MIN114 flow cell.

## ANNOUNCEMENT

*Leptothrix* are heterotrophic, relatively easy to grow, model metal-oxidizing bacteria from freshwater streams and wetlands (1–3). Cells are filaments in sheaths that bind oxidized metals (4). To increase the few *Leptothrix* isolates and genomes, we present two complete genomes from new isolates, *Leptothrix mechoopdaensis* BB-3 and *Leptothrix mechoopdaensis* BB-4.

**Fig 1:**
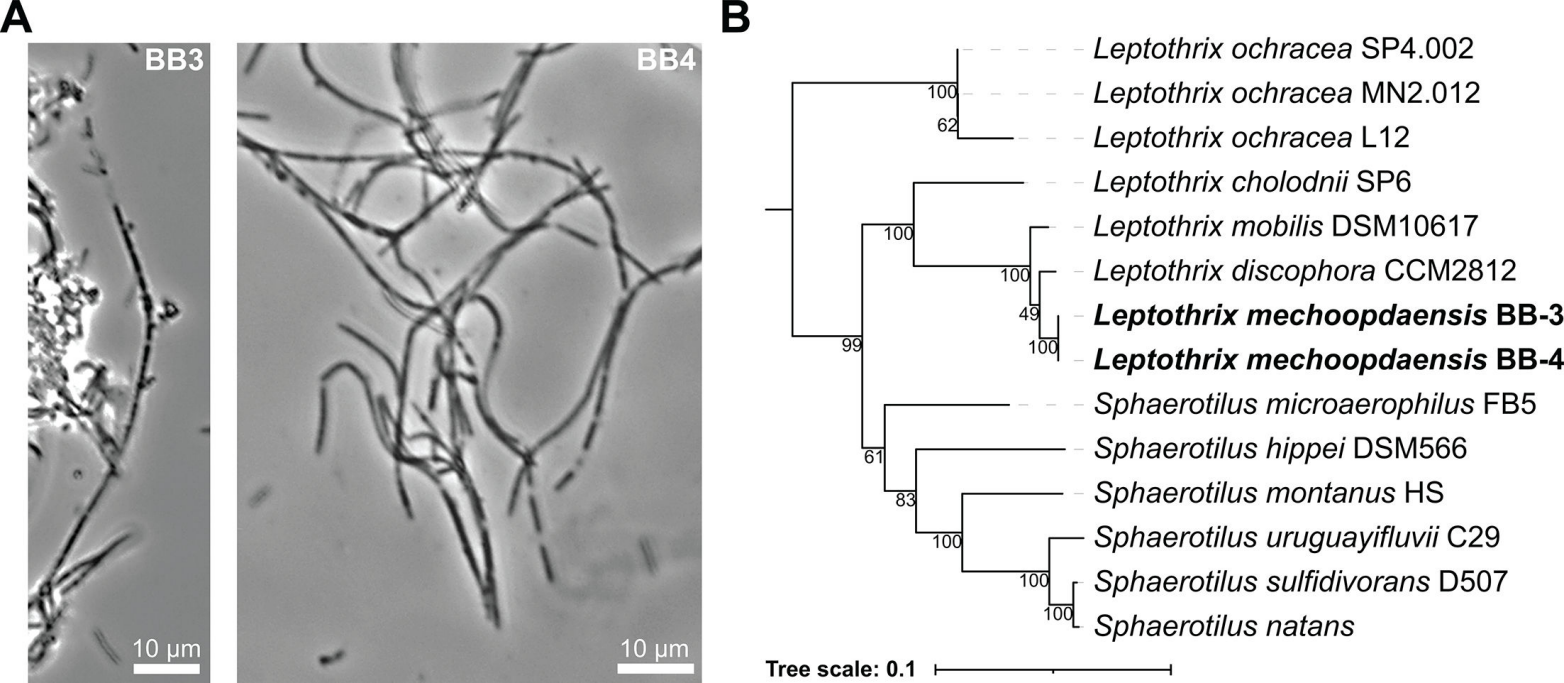
(**A**) Phase contrast microscopy images of *L. mechoopdaensis* BB-3 and BB-4 with streptobacilli morphology in a sheath. (**B**) Maximum likelihood tree of ribosomal proteins generated using RAxML with 1,000 bootstraps. *Rhodoferax ferrireducens* T118 outgroup not shown. Numbers indicate bootstrap support.

A flocculant mat was sampled in a wooded iron seep near Centerville, CA, USA (39° 48 14 N, 121° 38 47W), in October 2022, streaked onto modified Lepto media plates (5), and incubated at 27°C. Medium modifications were 3 g/L MnCO_3_ for MnCl_2_, MD-TMS trace element mixture (ATCC), and addition of 100 μL of 50 μM FeSO_4_ to the plate prior to streaking. After 5 days, colonies were dark, indicating metal oxidation; two were restreaked for purification. The BB-3 and BB-4 cell morphologies are streptobacilli within a sheath (Fig. 1A). The small subunit ribosomal DNA sequences were amplified using colony PCR with 8F and 1492R primers, which were 100% identical to one another and to *Leptothrix* spp. (Fig. 1B). Genomic DNA was isolated from cultures grown in minimal salts, vitamin, and pyruvate media (MSVP) (4) using the Qiagen DNeasy PowerSoil Pro Kit (Germany).

Sequencing libraries were prepared with the Rapid Barcoding Kit SQK-RBK 114.24 and sequenced with a FLO-MIN114 flow cell on the MinION Mk1B platform (Oxford Nanopore Technologies). Sequencing was monitored with the MinKNOW 24.02.6 interface and base calling (super accuracy, SUP, model dna_r10.4.1_e8.2_400bps_sup@v4.3.0). Adapter trimming and demultiplexing were performed with Dorado 0.5.3 (Oxford Nanopore Technologies). Demultiplexed files were converted to fastq using Samtools v.1.18.

Reads (619,186 for BB-3 and 361,331 for BB-4) were uploaded to the DOE Systems Biology Knowledgebase (KBase [6]). In Filtlong (7), reads < 1,000 bp or <5% of scores were discarded, with target bases at 1,500 Mbp. After filtering, 383,936 and 222,051 reads with N50s of 4,482 and 4,639 bp were obtained for BB-3 and BB-4, respectively. Reads were assembled with Flye v2.9.4 (Oxford Nanopore high-quality reads setting [8]) and yielded complete (estimated using CheckM (v1.0.18 [9]), circular genomes for BB-3 (length 5,069,072 bp; coverage 269×; 99.7%) and BB-4 (5,068,728 bp; coverage 160×; 100%).

Genes were called using Prodigal v2.6.3 (10) in Anvi’o v8 (11). Default settings were used unless otherwise indicated. Both have 4,385 genes, 69.70% GC content, and differ by 385 bases. A concatenated ribosomal protein phylogeny shows that the closest relatives are *Leptothrix discophora* and *Leptothrix mobilis* (Fig. 1B). Both genomes have 87.7% amino acid identity (AAI) to *L. discophora* and 87.8% AAI to *L. mobilis* (EzAAI v1.2.3 [12]). Both have 90.1% average nucleotide identity (ANI) to *L. discophora* and 89.6% ANI to *L. mobilis* (FastANI v0.1.3 [13, 14]). The strains differed from their closest relatives and were novel *Leptothrix* species (<95% species cutoff for ANI and AAI [15]). Both strains encode the Cluster 2 *cyc2* iron oxidase (FeGenie [16, 17]).

## Data Availability

Genome sequences for Leptothrix mechoopdaensis. BB-3 and BB-4 are deposited in the National Center for Biotechnology Information under bioproject PRJNA1336152; RefSeq GCF_053893855.1 and GCF_053893815.1; GenBank accession numbers GCA_053893855.1 and GCA_053893815.1; and raw reads SRX31475243 and SRX31475244 BB-3 and BB-4, respectively.

## References

[1] Mulder EG, Van Veenw S. 1963. Investigations on the sphaerotilusleptothrix group. Antonie Van Leeuwenhoek 29:121–153. doi:10.1007/BF02046045 14047145

[2] Spring S. 2006. The Genera Leptothrix and Sphaerotilus, p 758–777. In Subclasses Beta, Dworkin M, Falkow S, Rosenberg E, Schleifer KH, Stackebrandt E (ed), In the prokaryotes. Vol. 5. Springer New York: New York, NY.

[3] Emerson D, Fleming EJ, McBeth JM. 2010. Iron-oxidizing bacteria: an environmental and genomic perspective. Annu Rev Microbiol 64:561–583. doi:10.1146/annurev.micro.112408.134208 20565252

[4] Emerson D, Ghiorse WC. 1992. Isolation, cultural maintenance, and taxonomy of a sheath-forming strain of leptothrix discophora and characterization of manganese-oxidizing activity associated with the sheath. Appl Environ Microbiol 58:4001–4010. doi:10.1128/aem.58.12.4001-4010.1992 16348826 PMC183217

[5] Boogerd FC, de Vrind JP. 1987. Manganese oxidation by Leptothrix discophora. J Bacteriol 169:489–494. doi:10.1128/jb.169.2.489-494.1987 3804969 PMC211803

[6] Arkin AP, Cottingham RW, Henry CS, Harris NL, Stevens RL, Maslov S, Dehal P, Ware D, Perez F, Canon S, et al.. 2018. KBase: the United States department of energy systems biology knowledgebase. Nat Biotechnol 36:566–569. doi:10.1038/nbt.4163 29979655 PMC6870991

[7] Wick RR. 2017. Filtlong. Available from: https://github.com/rrwick/Filtlong

[8] Kolmogorov M, Yuan J, Lin Y, Pevzner PA. 2019. Assembly of long, error-prone reads using repeat graphs. Nat Biotechnol 37:540–546. doi:10.1038/s41587-019-0072-8 30936562

[9] Parks DH, Imelfort M, Skennerton CT, Hugenholtz P, Tyson GW. 2015. CheckM: assessing the quality of microbial genomes recovered from isolates, single cells, and metagenomes. Genome Res 25:1043–1055. doi:10.1101/gr.186072.114 25977477 PMC4484387

[10] Hyatt D, Chen G-L, Locascio PF, Land ML, Larimer FW, Hauser LJ. 2010. Prodigal: prokaryotic gene recognition and translation initiation site identification. BMC Bioinformatics 11:119. doi:10.1186/1471-2105-11-119 20211023 PMC2848648

[11] Eren AM, Kiefl E, Shaiber A, Veseli I, Miller SE, Schechter MS, Fink I, Pan JN, Yousef M, Fogarty EC, et al.. 2020. Community-led, integrated, reproducible multi-omics with anvi’o. Nat Microbiol 6:3–6. doi:10.1038/s41564-020-00834-3 PMC8116326 33349678

[12] Kim D, Park S, Chun J. 2021. Introducing EzAAI: a pipeline for high throughput calculations of prokaryotic average amino acid identity. J Microbiol 59:476–480. doi:10.1007/s12275-021-1154-0 33907973

[13] Jain C, Rodriguez-R LM, Phillippy AM, Konstantinidis KT, Aluru S. 2018. High-throughput ANI Analysis of 90K Prokaryotic Genomes Reveals Clear Species Boundaries. Bioinformatics. Bioinformatics, Bioinformatics. doi:10.1101/225342 PMC6269478 30504855

[14] Goris J, Konstantinidis KT, Klappenbach JA, Coenye T, Vandamme P, Tiedje JM. 2007. DNA-DNA hybridization values and their relationship to whole-genome sequence similarities. Int J Syst Evol Microbiol 57:81–91. doi:10.1099/ijs.0.64483-0 17220447

[15] Konstantinidis KT, Tiedje JM. 2007. Prokaryotic taxonomy and phylogeny in the genomic era: advancements and challenges ahead. Curr Opin Microbiol 10:504–509. doi:10.1016/j.mib.2007.08.006 17923431

[16] Garber AI, Nealson KH, Okamoto A, McAllister SM, Chan CS, Barco RA, Merino N. 2020. FeGenie: a comprehensive tool for the identification of iron genes and iron gene neighborhoods in genome and metagenome assemblies. Front Microbiol 11:37. doi:10.3389/fmicb.2020.00037 32082281 PMC7005843

[17] McAllister SM, Polson SW, Butterfield DA, Glazer BT, Sylvan JB, Chan CS. 2020. Validating the Cyc2 neutrophilic iron oxidation pathway using meta-omics of Zetaproteobacteria iron mats at marine hydrothermal vents. mSystems 5:e00553-19. doi:10.1128/mSystems.00553-19 PMC7029218 32071158

